# Topical dual-probe staining using quantum dot-labeled antibodies for identifying tumor biomarkers in fresh specimens

**DOI:** 10.1371/journal.pone.0230267

**Published:** 2020-03-11

**Authors:** Boyu Meng, Margaret R. Folaron, Brook K. Byrd, Kimberley S. Samkoe, Rendall S. Strawbridge, Connor Barth, Summer L. Gibbs, Scott C. Davis

**Affiliations:** 1 Thayer School of Engineering at Dartmouth College, Hanover, New Hampshire, United States of America; 2 Geisel School of Medicine at Dartmouth College, Hanover, New Hampshire, United States of America; 3 Department of Surgery, Dartmouth-Hitchcock Medical Center, Lebanon, New Hampshire, United States of America; 4 Norris Cotton Cancer Center, Dartmouth-Hitchcock Medical Center, Lebanon, New Hampshire, United States of America; 5 Biomedical Engineering Department, Oregon Health and Science University, Portland, Oregon, United States of America; University of California Berkeley, UNITED STATES

## Abstract

**Purpose:**

Rapid, intra-operative identification of tumor tissue in the margins of excised specimens has become an important focus in the pursuit of reducing re-excision rates, especially for breast conserving surgery. Dual-probe difference specimen imaging (DDSI) is an emerging approach that uses the difference in uptake/clearance kinetics between a pair of fluorescently-labeled stains, one targeted to a biomarker-of-interest and the other an untargeted isotype, to reveal receptor-specific images of the specimen. Previous studies using antibodies labeled with either enhanced Raman particles or organic fluorophores have shown promising tumor vs. normal diagnostic performance. Yet, the unique properties of quantum dot-labeled antibody complexes (QDACs), which provide spectrally-distinct fluorescence emission from a common excitation source, make them ideal candidates for this application. Herein, we evaluate the diagnostic performance of QDAC-based DDSI in excised xenografts.

**Procedures:**

Excised fresh specimens of normal tissue and human tumor xenografts with elevated expression of HER2 were stained with a HER2-targeted QDAC and an untargeted QDAC isotype. Stained specimens were imaged on a custom hyperspectral imaging system capable of spectrally separating the quantum dot signatures, and images processed using the DDSI approach. The diagnostic performance of this technique under different incubation temperatures and probe concentrations was evaluated using receiver-operator characteristic analysis.

**Results:**

HER2-targeted QDAC-DDSI was able to distinguish HER2(+) tumors from normal tissue with reasonably high diagnostic performance; however, this performance was sensitive to temperature during the staining procedure. Area under the curve values were 0.61 when staining at room temperature but increased to over 0.81 when staining at 37 °C. Diagnostic performance was not affected by increasing stain concentration.

**Conclusions:**

This study is the first to report dual-probe difference imaging of specimens using QDACs and hyperspectral imaging. Our results show promising diagnostic performance under certain conditions, and compel further optimization and evaluation of this intra-operative margin assessment technique.

## Introduction

Incomplete removal of tumor during breast conserving surgery (BCS) is a widely-recognized problem that affects a large percentage of patients undergoing this procedure [1, 2]. A diagnosis of tumor-involved margins is typically determined by pathological analysis of the surgical specimen days after the surgery, and often triggers a costly second surgery which is a tremendous burden to patients, elevates risk of morbidity, and delays adjuvant therapy [1, 3, 4]. Shifting this diagnostic determination to occur during the primary surgery, when additional tissue can still be removed, should improve primary surgery outcomes and reduce the rates of secondary surgery. Although techniques such as touch prep cytology, frozen section analysis, specimen radiography or ultrasonography, among others, are currently available for intra-operative margin assessment, none have fully addressed the issue due to some combination of impracticality, loss of tissue for follow-up analysis or inadequate diagnostic performance [5]. Recognizing the inadequacy of these techniques, widespread research efforts are underway to develop new technologies to improve primary surgery outcomes. A substantial proportion of these efforts aim to leverage endogenous or exogenous optical contrast in the surgical cavity or excised specimens [6–26]. Although many of these modalities have shown promise and are in various stages of clinical translation, a leading candidate that dramatically reduces the re-excision rates in BCS has yet to emerge as a standard of care.

Among the optical approaches under investigation for this application are techniques designed to stain and image the excised specimens for intra-surgical assessment of specimen margins. These strategies can take a variety of forms, including the use of enzyme-activated fluorescent probes [27, 28], nuclear staining with microscopy to recapitulate histopathological analysis [16, 29, 30], or staining of tumor biomarkers with molecular-probes for wide-field imaging [20, 31–33]. The latter modality has been shown to be most effective when applied using dual-probe techniques which involve incubating the specimen in a solution consisting of two spectrally-distinct optical probes; one that is specific to the target of interest, and an untargeted isotype, before imaging. The images of each probe are then processed in a manner that corrects for instrument inhomogeneities and helps account for non-specific uptake of the targeted probe to emphasize or quantify the biomarker(s) of interest. These normalization/correction procedures can involve simple subtraction, ratio calculation, or more sophisticated model-based methods to provide quantitative information about receptor engagement [34–36]. Experimentally, dual-probe techniques have been applied using antibody-labeled surface-enhanced Raman spectroscopy particles, which allow multiplexing of multiple targets of interest due to the fine spectral features of the Raman signatures [19, 31, 32], or antibodies labeled with conventional organic fluorophores [20, 37].

Prior development of dual-probe difference specimen imaging in our lab (which we term DDSI) has focused on the use of antibodies labeled with conventional fluorophores deployed with two-channel wide-field fluorescence imaging. Although these probe pairs have shown very promising diagnostic performance in animal models (area under the curve [AUC] of receiver operating characteristic [ROC] curves = 0.97), the broad excitation and emission profiles of the labels requires careful choice of excitation and filter settings, and it’s often the case that multiple excitation sources are required. Multiplexing sources for this application contribute to longer imaging times and can exacerbate the confounding effects of wavelength-dependent optical properties of the tissue. This is a particular challenge for sources with dramatically different wavelengths, as would be required to increase the number of unique spectral probes, and thus targeted biomarkers, used in the staining protocol.

The use of quantum dot antibody conjugates (QDACs) for dual-probe difference specimen imaging (QDAC-DDSI) is an attractive option which addresses the aforementioned challenges. Choosing from catalogues of quantum dots that produce spectrally-distinct fluorescence emission yet share an excitation profile allows excitation of a cocktail of probe pairs with a single excitation source [38], and thus a common excitation volume. Leveraging this property by introducing hyperspectral imaging can enable two or more probe pairs to be used to examine multiple biomarkers simultaneously, an important capability for accommodating heterogeneous cell phenotypes commonly found in tumors. Furthermore, these particles also generally have high quantum yield and are photostable, both advantageous characteristics for deploying the technique in the clinic. While prior studies have shown QDAC staining of ex-vivo tissues with a single probe in animal models [33], to our knowledge these complexes have not been deployed in the DDSI paradigm.

In this report, we describe the development and evaluation of hyperspectral QDAC-based DDSI of fresh specimens. Cell culture experiments were used to confirm specific binding and assess non-specific uptake of the QDACs in a cell line with elevated receptor expression as well as a comparable negative control. Next, we used a custom-built wide-field hyperspectral imaging system to evaluate the performance of HER2-targeted QDAC-DDSI using tumor xenografts with elevated expression of HER2 and normal tissue under various staining conditions. Specifically, we examined the effect of; (1) staining incubation temperature, (2) staining solution concentration and (3) quantum dot channel, in which the quantum dot labels were switched between targeted and untargeted antibodies. In each case, ROC analysis of the resulting images was used to assess the performance of the protocol and recommend future development of this multiplexed, topical staining approach to margin assessment.

## Materials and methods

### Quantum dot-antibody complex (QDAC) preparation

The DDSI imaging strategy uses a receptor-targeted fluorescent reporter and a spectrally-distinct untargeted counterpart. In this study, the targeted probe consisted of approximately 20 nm quantum dots (either QD-605 or QD-655, Thermo Fisher Scientific, Waltham MA) conjugated to the anti-HER2 antibody, trastuzumab (Herceptin, Genentech, South San Francisco, CA). Note that herein, we refer to non-conjugated quantum dots as QD. The untargeted probe consisted of QD-605 or QD-655 conjugated to the untargeted complex, mouse-IgG (Thermo Fisher Scientific, Waltham MA). In all cases, quantum dot-antibody conjugation was performed using the SiteClick conjugation kit. This process involves modifying the Fc region on the antibody prior to attachment of the DIBO-labeled quantum dot. QDACs were wrapped in aluminum foil and stored at 4°C. Because the absorbance spectra of quantum dots conceal the absorbance of proteins at 280 nM, the concentration of the quantum dot in the complex was used to determine QDAC concentration.

### Cell lines and flow cytometry

The MCF/HER2-18 cells (here termed HER2(+) cells) were originally developed by and reported on by Benz CC et al. [39]. This cell line, along with the parent MCF-7 line (ATCC, here termed HER2(-) cells) were kindly provided by Dr. Maximus Kullberg at the University of Colorado, Denver to Dartmouth College in 2012. Prior to conducting cell and animal experiments in this study, flow cytometry was used to determine HER2 expression levels in the two breast cancer cell lines. Cell preparation is described in detail in S1 Appendix. Quantification of HER2 expression was performed through comparison of Alexa Fluor 488-trastuzumab stained cells to Quantum Cy5 MESF Beads (Bangs Laboratory, Fishers, IN), as previously described [40]. Analysis was performed using the Cell Quest Acquisition software.

### Confirmation of QDAC-receptor specificity using cell staining

Cell culture studies in the HER2(+) and HER2(-) cell lines were completed to confirm specific-receptor binding of targeted QDACs and assess nonspecific binding of untargeted QDACs. Specifically, each cell line was incubated with every combination of the following QDACs: QDAC-655-targeted, QDAC-605-targeted, QDAC-655-untargeted and QDAC-605-untargeted. The cells were maintained in DMEM culture media supplemented with 5% fetal bovine serum and 1% 1X Penicillin-Streptomycin at 5% CO_2_ and 37 °C. For staining evaluation, approximately 5x10^4^ MCF-7-parent or MCF-7-HER2 cells were plated in a black-walled clear-bottom 96 well plate and then washed with PBS and 2% paraformaldehyde. Cells were then submerged in a blocking solution of 10% horse serum for 30 minutes. After 30 minutes, a 30 nM solution of QDACs were added to the blocking solution and incubated the cells for two hours, followed by a series of PBS washes. In addition, cells were incubated for 5 minutes with 4′, 6-diamidino-2-phenylindole (DAPI) counterstain (Thermo Fisher Scientific, Waltham, MA). Stained cells were immediately imaged using an Olympus BX51 inverted fluorescence microscope. QDAC staining in each image was quantified by computing the summed stain intensity above the noise floor threshold using ImageJ V2.0. Reported values were normalized to the highest signal intensity from all eight images.

### Animal use protocols

All experiments involving animals were reviewed and approved by the Institutional Animal Care and Use Committee (IACUC) at Dartmouth College and conducted in accordance with IACUC protocols and the 1964 Helsinki declaration. Animals were purchased from Charles River Laboratories, Inc. (Wilmington, MA) and housed in standard laboratory housing containing sheltering objects. A total of seven animals were used I this study. Environmental enrichment was provided in the form of brown crinkle paper in each cage and animals were provided ad libitum access to food and water. All surgical procedures (pellet and tumor implantation) were conducted on fully anesthetized animals. Animals were euthanized using cervical dislocation while under deep anesthesia.

### Tumor xenografts

Three days prior to tumor cell implantation, an estrogen pellet (0.72mg/90 day release, Innovative Research of America, Sarasota, FL) was placed subcutaneously in the back of the neck of female Nu/Nu mice. Mice were injected subcutaneously in the lower dorsal region on both sides with 1x10^6^ MCF-7-HER2 cells suspended in 50 μL of Matrigel (Corning Inc., Tewksbury, MA) and monitored for recovery following the procedure. Two tumors grew on each mouse and a total of seven animals were used in the study. Once tumors reached a pinch-measurement length of 1 cm, mice were euthanized and tumor and muscle samples were resected and cut into 4–7 mm thick slices in preparation for staining. As has been reported in prior studies, only the cut surface of tumor slices were used for imaging.

### Quantum-dot antibody conjugate-based dual-stain difference specimen imaging (QDAC-DDSI) of fresh specimens

**Fig 1:**
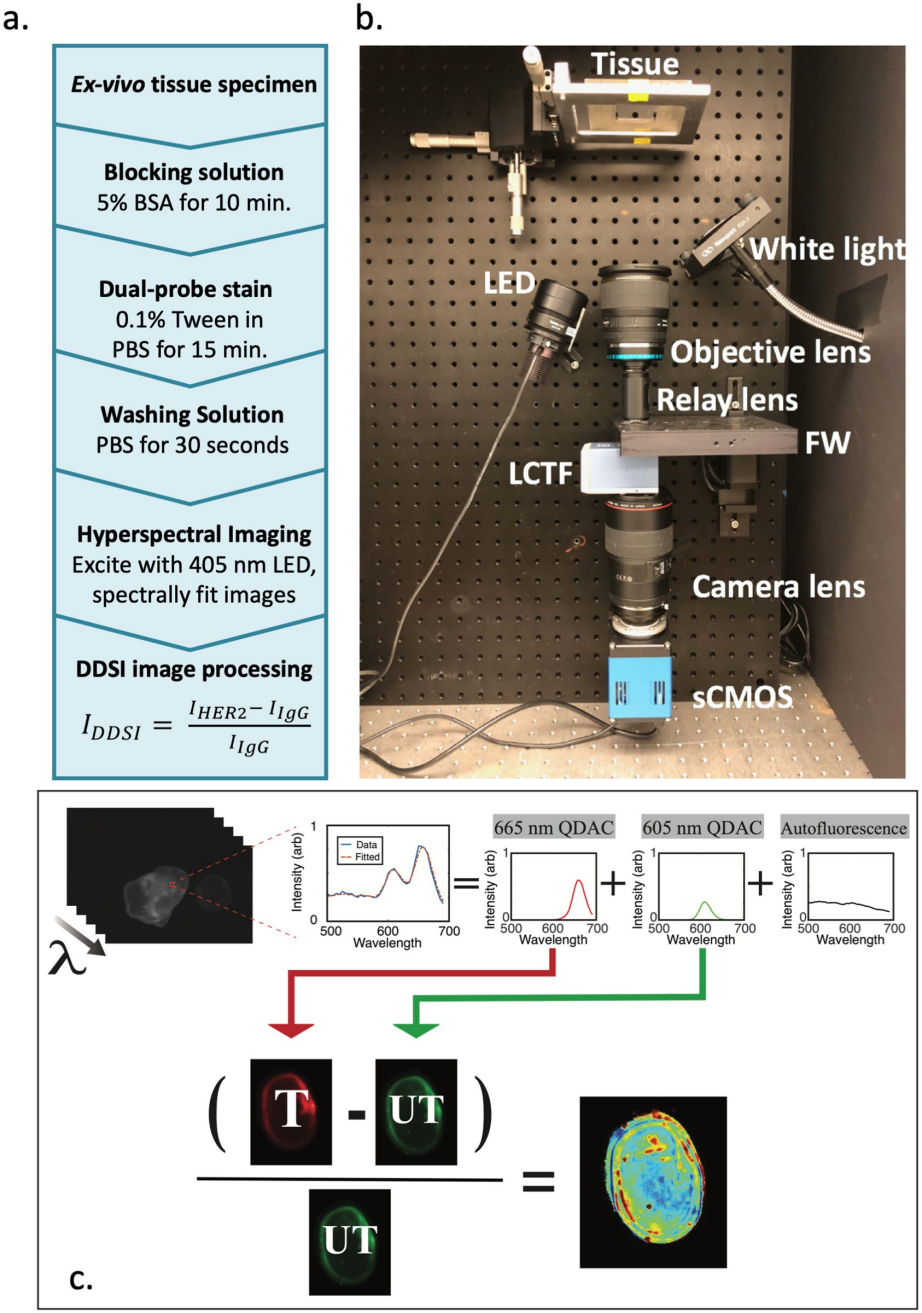
(a) Schematic of the QDAC-DDSI tissue staining protocol. (b) Photograph of the hyperspectral specimen imaging system with major components labeled. (c) Schematic of the image processing steps. Pixel-wise spectral fitting of the hyperspectral image stack extracts the two QDAC channels, which are then normalized to a calibration volume and used to compute the DDSI image.

A schematic of the tissue staining and imaging protocol is provided in Fig 1(a), and similar protocols for non-quantum dot DDSI have been described previously [20, 37]. In this study, specimens were first submerged in a blocking solution consisting of 5% bovine serum albumin (BSA) for 10 minutes. Following blocking, tissues were incubated for 15 minutes in the staining solution, consisting of equal concentrations of targeted and untargeted QDACs in PBS with 0.1% Tween. After stain incubation, a 30 second wash in PBS was performed. Tissues were then placed on glass slides for imaging with the DDSI hyperspectral imaging system, accompanied by a well of the original staining solution for image calibration.

The tissue staining experiments were designed to evaluate the diagnostic performance of QDAC-based DDSI under different temperature, concentration, and labeling conditions in HER2(+) (MCF-7 transfected with HER2) xenografts. Specifically, we computed the ROC metrics to compare the following conditions (N = 4–6 tumor-normal tissue pairs for each condition):

1. Temperature: QDAC-655-untargeted/QDAC-605-targeted staining at room temperature (22°C) vs. 37°C.
2. Stain concentration: QDAC-655-untargeted/QDAC-605-targeted staining at concentrations of 10 nM vs. 100 nM.
3. Label: QDAC-655-untargeted/QDAC-605-targeted staining vs. QDAC-605-untargeted/QDAC-655-targeted staining

The 37°C temperature was maintained by keeping stains in a cell incubator before and during specimen incubation and room temperature staining was performed on a benchtop in a highly environmentally-controlled lab. The sample number breakdown was as follows: 10 nM, 37°C group: N = 4 (two tumors from two mice), 10 nM, 22°C group: N = 4 (two tumors from two mice), 100 nM group: N = 6 (six tumors from four mice), and reversed label group: N = 5 (five tumors from three mice).

### Hyperspectral DDSI imaging system and image processing

The imaging system, pictured in Fig 1(b), is a custom-built broad-beam epi-illumination instrument with hyperspectral detection. The system is configured to image from the bottom of a transparent plate upon which the samples are positioned. For the QDAC-DDSI imaging reported herein, tissue samples were illuminated with a slightly off-axis 405 nm LED light source (Thorlabs M405L2, Newton, NJ) collimated with an aspheric condenser lens. The illuminated area was set to 21 mm by 21 mm at 155.7 mW/cm^2^. Fluorescence light emitted from the samples was collected with a 24 mm camera lens (Sigma, Ronkonkoma, NY) and then passed through a relay lens configuration containing a high-speed motorized filter wheel and VariSpec VIS liquid crystal tunable filter (LCTF, PerkinElmer, Waltham, MA) before being focused on a PCO.Edge 4.2 scientific CMOS camera (Kelheim, Germany). For this application, the motorized filter wheel positioned a 480 long-pass dichroic filter in the detection channel to substantially reduce excitation bleed-through before filtering with the LCTF. The tunable filter acquired images from 500 nm to 700 nm in increments of 5 nm to produce spectral image stacks with high spatial (55 μm, determined using the USAF1951 test target) and spectral resolution. Total image acquisition times were around 60 seconds for these 40 wavelengths.

The hyperspectral imaging configuration enables spectral de-coupling of multiple fluorescence signatures at each pixel. To accomplish this de-coupling, the pure fluorescence spectra of QD-605, QD-655 and tissue autofluorescence were pre-recorded as basis spectra. When specimens were imaged, these basis spectra were used in a linear-least-squares spectral fitting routine applied pixel-by-pixel to the acquired hyperspectral imaging stack. An example of acquired spectra and the basis fits is provided in Fig 1(c). This process collapses the hyperspectral data into three images: the relative intensity of each quantum dot channel (QDAC-605 and QDAC-655) and tissue autofluorescence.

The post-spectral-fitting QDAC-605 and QDAC-655 images were used to determine the DDSI image maps. First, the image of each channel was normalized to the mean value of fluorescence intensity in the calibration volume. After normalization, the DDSI value ((targeted-untargeted)/untargeted) was calculated to produce the DDSI image maps, as illustrated in Fig 1(c). As previously reported [37], ROC curves were determined using the perfcurve function in Matlab on a pixel-by-pixel basis, with truth defined by manually tracking harvested tumor and normal specimens through the staining process. Area under the curve (AUC) were values extracted to compare diagnostic performance of each condition.

### Immunohistochemical (IHC) staining and microscopy

After topical tissue staining and DDSI was completed, each specimen was placed in 10% formalin (Biochemical Science Inc, Swedesboro, NJ) for at least 24 hrs and then prepared for IHC staining of HER2. Four micron slices were cut and mounted on Leica Bond Plus Slides (Cat # 00270) and air-dried at room temperature. Using the automated protocol of the Leica Bond Rx Automated Stainer (Leica Products/Equipment, Leica Microsystems, Inc., Buffalo Groove, IL), the slides were baked for 30 minutes and dewaxed with Leica Bond Dewax solution (Cat #AR9222). The antigen retrieval was Bond Epitope Retrieval 2 (Cat #ar9640), carried out in a pH 9.0 solution for 20 minutes. The HER2 primary antibody dilution was 1:300 for 15 minutes (Abcam Cat # ab16901; Abcam Inc., Cambridge, MA). Primary antibody binding was visualized using Leica Bond Refine Detection kit (Cat # DS9800) with a diaminobenzidine (DAB) chromogen and a hematoxylin counterstain. Bright field, whole fit images of stained tissue (H&E and HER2 IHC) were scanned using the PerkinElmer Vectra3 slide scanner.

## Results

### Confirming linearity of response to quantum dot concentration

**Fig 2:**
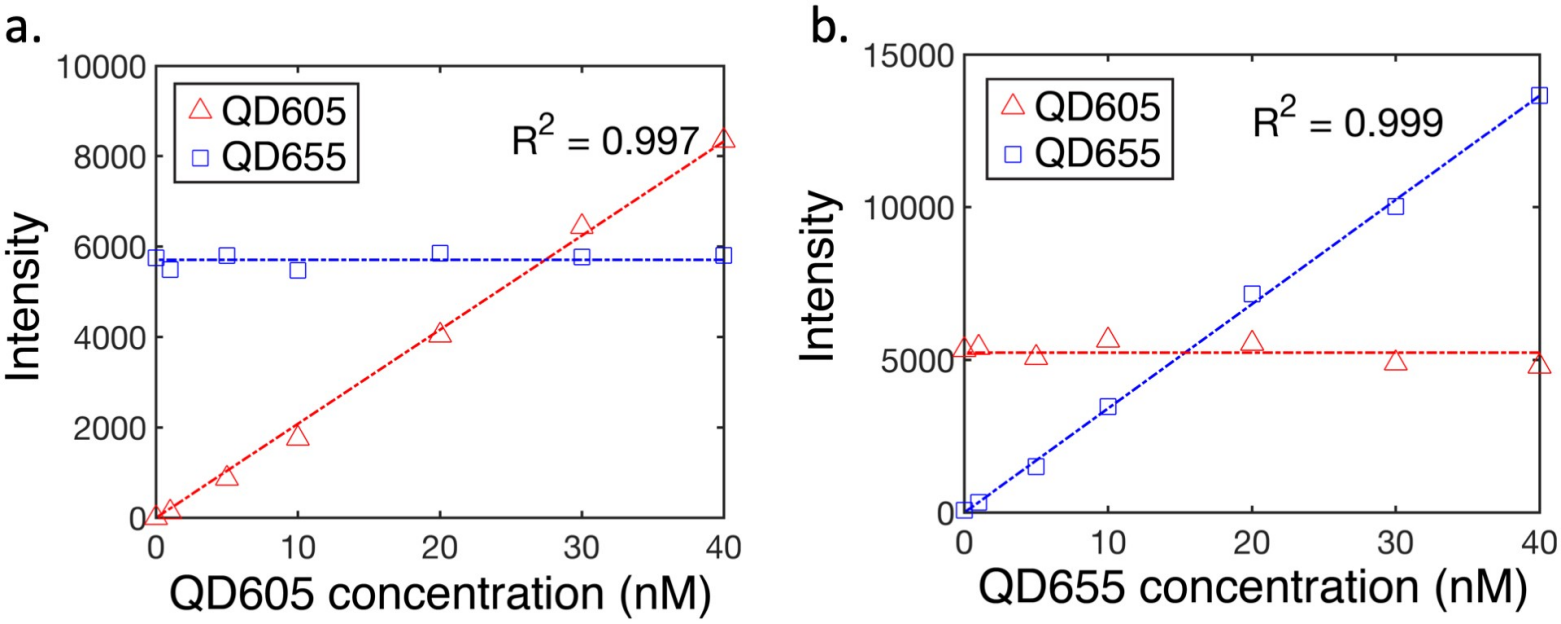
Linearity of response of the hyperspectral imaging system to co-localized quantum dot concentration in tissue-simulating phantoms: (a) Mean values of fluorescence signal plotted as a function of QD-605 concentration (0 to 40 nM) while QD-655 was maintained at 20 nM. In (b), similar data for the reverse case (QD-605 constant with changing QD-655) are shown.

To confirm the approach provides a linear and independent response to quantum dot concentration, we imaged tissue-simulating liquid phantoms containing co-localized quantum dot solutions of varying concentrations. Specifically, we prepared phantom solutions containing 1% intralipid in PBS, and varying concentrations of QD-605 and QD-655. In one series, the concentration of QD-605 was held constant (20 nM) the concentration of QD-655 was varied between 0 and 40 nM (0, 1, 5, 10, 20, 30 and 40 nM). This protocol was then repeated holding QD-655 constant (20 nM) and varying QD-605 over the same range. Each condition was imaged with the hyperspectral DDSI system and images processed as described in the Methods section. Mean values of fluorescence intensity from each channel for both series are plotted in Fig 2. These results show a highly-linear response to changing concentration (R^2^>0.99 in both cases) with no discernable inter-channel crosstalk.

### QDACs show receptor-specific staining patterns in cell culture

**Fig 3:**
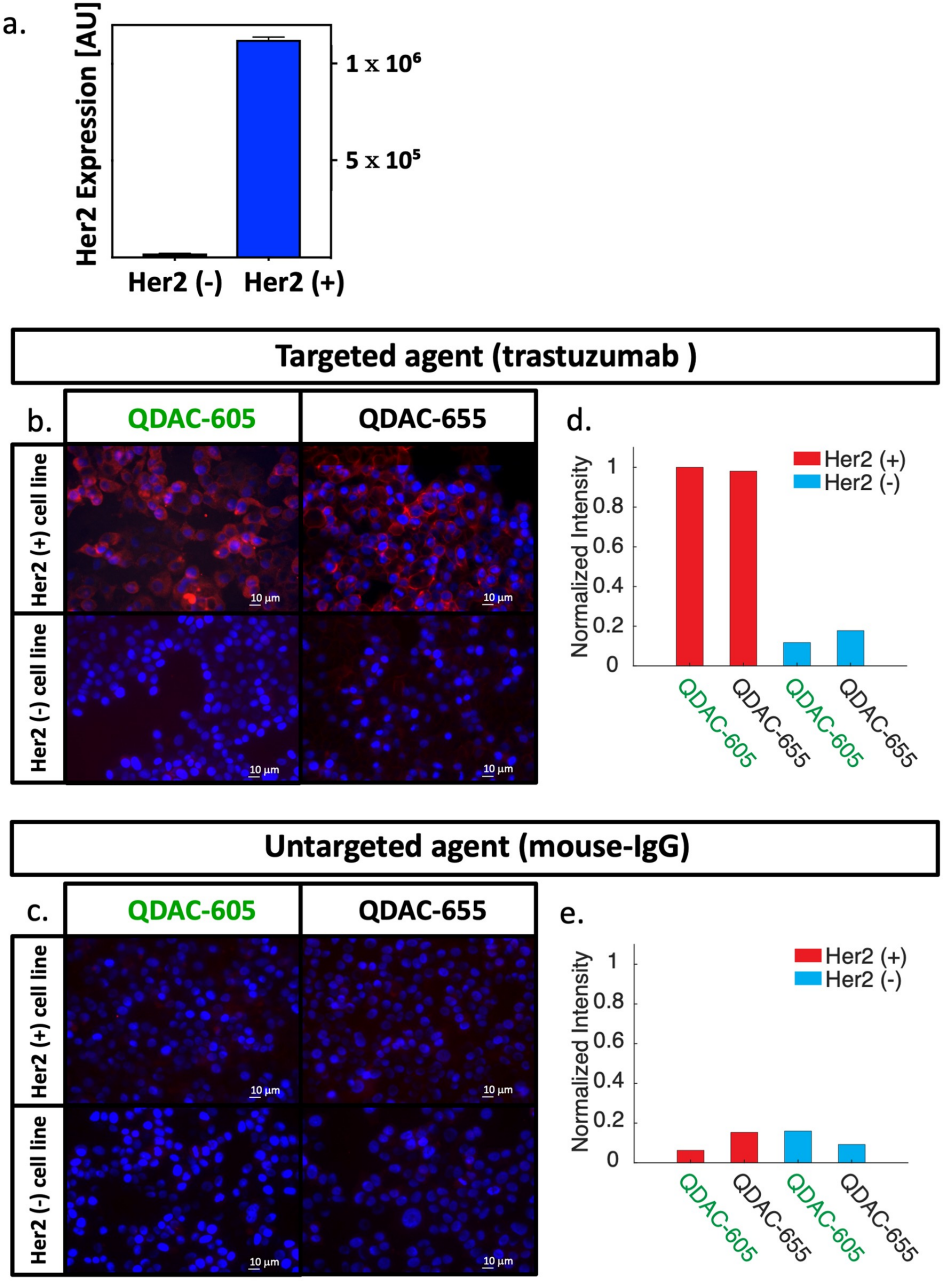
Confirmation of receptor-specific binding in vitro: (a) HER2 expression of two breast cancer cell lines (MCF7-parent line, termed HER2(-) and a transfected MCF7 line expressing HER2, termed HER2(+)), determined by flow cytometry. (b) Representative fluorescence micrographs of HER2(+) and HER2(-) cells stained in vitro with QDAC-605-targeted (QD-605 conjugated to trastuzumab) and QDAC-655-targeted (QD-655 conjugated to trastuzumab). The red channel represents quantum dot fluorescence and blue DAPI staining. (c) Fluorescence micrographs of both cell lines stained with the untargeted QDAC counterparts (QD-605 or QD-655 conjugated to mouse-IgG). Scale bars on all images are 10 μm. Quantitative analysis of images in (b) and (c) are provided in (d) and (e), respectively.

Fig 3(a) shows the flow cytometry results for HER2 expression in the HER2(+) and HER2(-) cell lines, confirming that the HER2 expression levels in the transfected line are substantially elevated compared to the parent line. Fig 3(b) and 3(c) show fluorescence microscopy images of plated cells after incubation with the targeted or untargeted QDACs, along with corresponding DAPI-stained images (in blue). Fig 3(b) shows both cell lines incubated with either of the HER2-targeted conjugates: QDAC-655-targeted or QDAC-605-targeted. The images for both targeted conjugates show robust fluorescence signal in the HER2(+) cell line, consistent with transmembrane-receptor staining, yet minimal fluorescence signal in the HER2(-) (MCF-7-parent) line. These results, confirmed by the quantitative analysis shown in Fig 3(d) suggest the targeted QDACs are specific to HER2 in cell culture. Fig 3(c) shows the two cell lines incubated with the untargeted conjugates based on mouse-IGg: QDAC-655-untargeted or QDAC-605-untargeted. The corresponding quantitative analysis is provided in Fig 3(e). Quantum dot fluorescence of these untargeted QDACs was minimal in both cell lines and quantum-dot channels, confirming that the untargeted conjugates have minimal non-specific uptake in these tumor cells.

### Stain incubation temperature affects QDAC-DDSI performance

**Fig 4:**
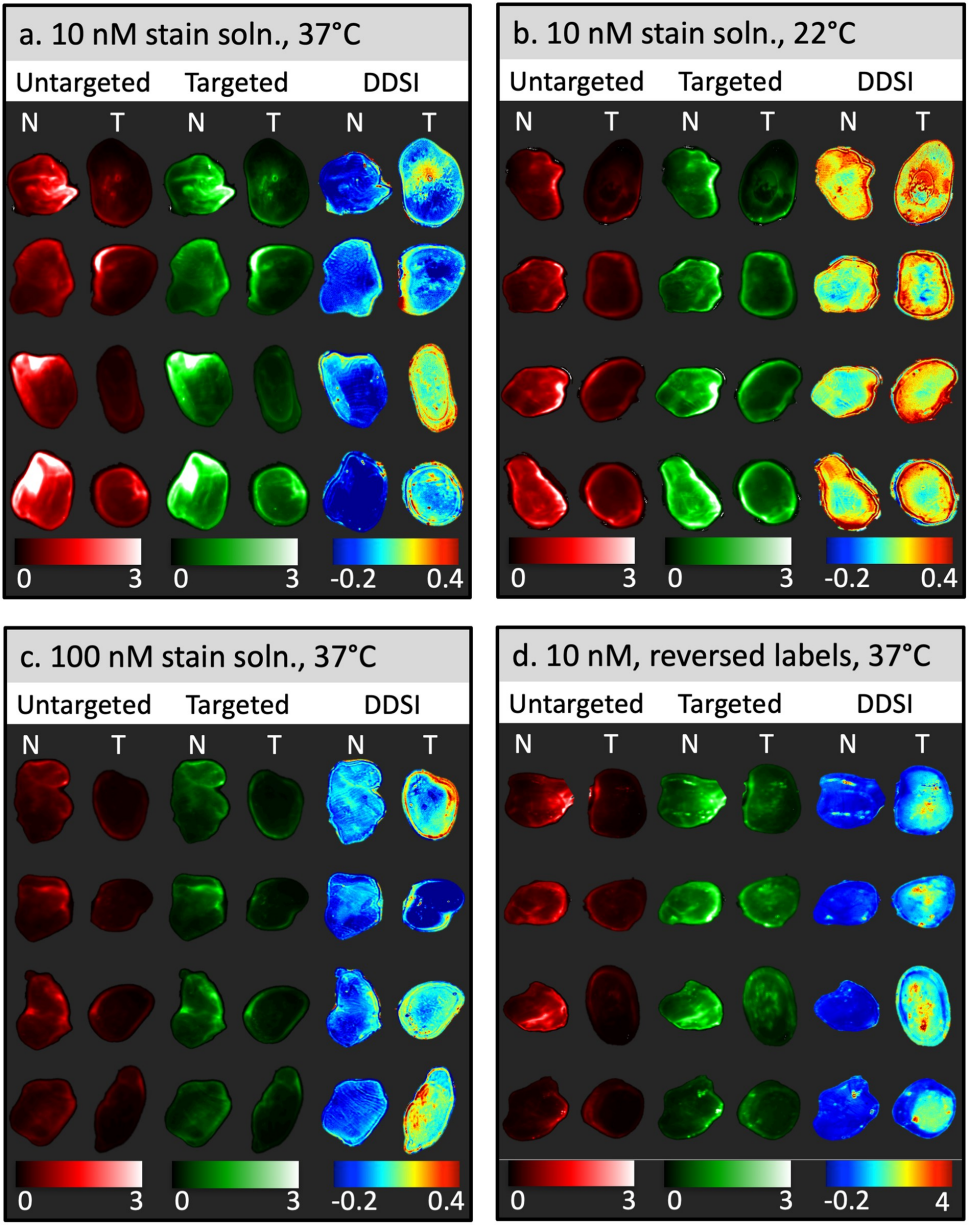
Representative specimen images after topical staining/washing for all four staining conditions investigated. Columns labeled “Untargeted” and ‘Targeted” represent images of the tissue after pixel-by-pixel spectral fitting of the hyperspectral data and normalization to the calibration volume, and thus show the fluorescence of the targeted and untargeted QDAC channels. The DDSI column shows the processed DDSI images. “N” and “T” refer to normal and tumor tissue, respectively. Each panel presents four specimen samples from each staining condition: (a) Incubation in a 10 nM stain solution at 37 °C, (b) Incubation in a 10 nM stain solution at 22 °C, (c) Incubation in a 100 nM stain solution at 37 °C, (d) Incubation in a 10 nM stain solution at 37 °C with the quantum dot labels reversed.

The performance of QDAC-based DDSI under different stain incubation temperatures was evaluated using fresh MCF-7-HER2 xenografts and accompanying normal tissue imaged with the hyperspectral DDSI imaging system described above. Images of fluorescence from the targeted and untargeted QDACs (after spectral fitting), as well as the DDSI parameter in normal and tumor tissues are shown in Fig 4(a) and 4(b) for 37 °C and 22 °C incubation temperatures, respectively. Inspection of the untargeted and targeted channel images alone suggests that these single-stain channels have no capacity to identify tumor from normal tissue. In fact, in most specimens, the normal tissue produced a higher signal than in the tumor, even in the targeted channel, suggesting non-specific uptake is a dominant contributor to the signal.

**Fig 5:**
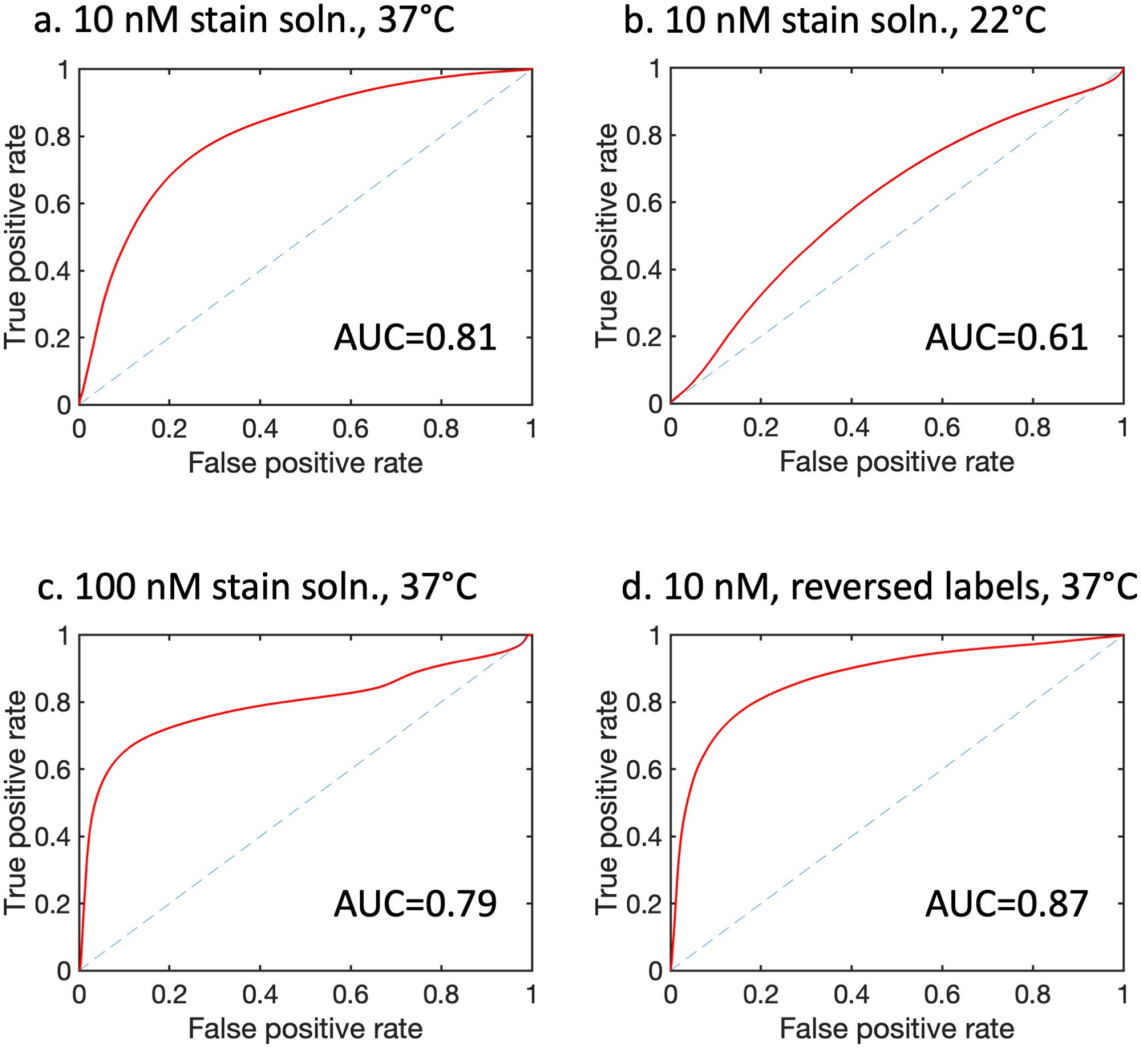
Receiver operating characteristic curves and AUC values plotted for each condition corresponding to the panel arrangement in Fig 4: (a) Incubation in a 10 nM stain solution at 37 °C (N = 4 tumor specimens), (b) Incubation in a 10 nM stain solution at 22 °C (N = 4 tumor specimens), (c) Incubation in a 100 nM stain solution at 37 °C (N = 6 tumor specimens), (d) Incubation in a 10 nM stain solution at 37 °C with the quantum dot labels reversed (N = 5 tumor specimens).

The corresponding DDSI parameter was sensitive to the stain incubation temperature. Qualitative inspection of the images in Fig 4(a) and 4(b) indicates that QDAC-DDSI was unable to distinguish between tumor and normal tissue when incubated at room temperature. Conversely, the higher temperature incubation condition produced images more concordant with receptor expression. ROC curves for the two conditions are plotted in Fig 5(a) and 5(b), and show a significant difference in diagnostic performance depending on incubation temperature. Room temperature QDAC-DDSI resulted in an AUC of 0.61 while application of QDAC-DDSI at 37 °C improved the AUC to 0.81.

**Fig 6:**
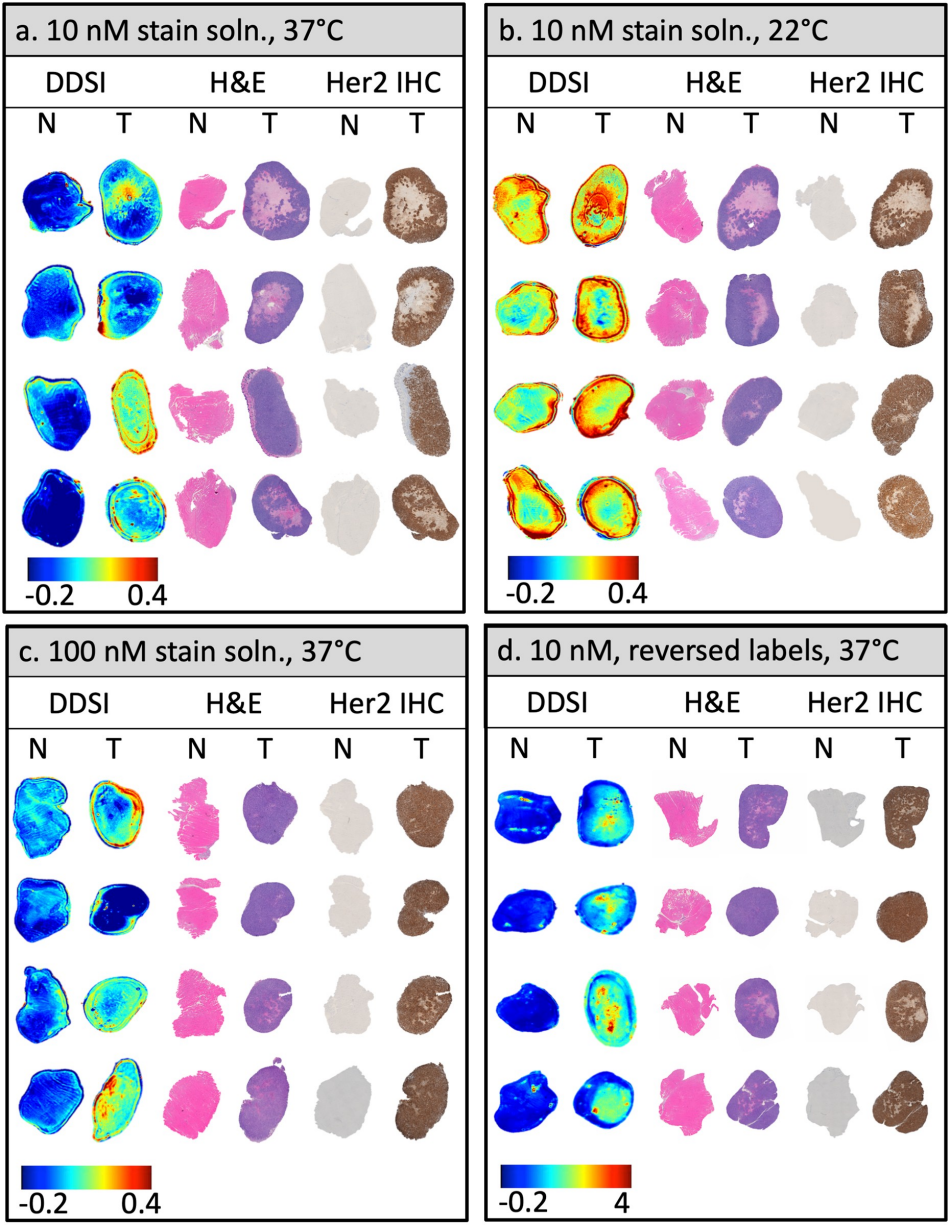
Images of DDSI, H&E and HER2-IHC for each tissue specimen for all staining conditions investigated: (a) Incubation in a 10 nM stain solution at 37 °C, (b) Incubation in a 10 nM stain solution at 22 °C, (c) Incubation in a 100 nM stain solution at 37 °C, (d) Incubation in a 10 nM stain solution at 37 °C with the quantum dot labels reversed. DDSI images represent the surface of fresh specimens while H&E and HER2-IHC are from tissue sections as close to the surface as possible.

The DDSI fluorescence staining patterns of tissue from both temperature conditions were examined with histological and HER2-IHC slides taken from approximately the same plane that the bulk tissue was imaged, shown in Fig 6(a) and 6(b). The visual correlation of DDSI maps and IHC HER2 expression was weak in both tumor and muscle tissue in the room temperature cohort. The muscle tissue showed high intensity in the DDSI maps in 3 out of 4 samples, although the corresponding HER2 IHC exhibited no expression of the HER2 receptor. Conversely, the high signal intensity of DDSI maps in the 37° temperature cohort demonstrated better visual correlation with areas of high signal intensity and regions of high IHC HER2 expression in tumor sections. No tissue degradation was observed in the higher temperature cohort.

### QDAC stain concentration did not affect QDAC-DDSI performance

To investigate the effects of stain concentration on QDAC-DDSI, resected tumor and normal tissue were processed with either a 10 nM or 100 nM stain solution. All other conditions were maintained and the staining was performed at 37 °C. Fig 4(c) shows the untargeted, targeted and DDSI images of the tissue specimens for the 100 nM staining protocol. Qualitative comparison of the DDSI images between the 10 nM and 100 nM stain solutions reveals no obvious difference between the two conditions. This is confirmed by the quantitative ROC analysis presented in Fig 5(c), which shows a slightly higher AUC value for the 10 nM concentration (AUC = 0.81) compared to the 100 nM concentration (AUC = 0.79). The DDSI images are plotted with the corresponding H&E and HER2 IHC in Fig 6(c). Although the patterns loosely follow the receptor density in many samples, this qualitative correlation is not robust in all tissues.

### Quantum dot label switching showed a modest increase in QDAC-DDSI performance

QDAC-DDSI was repeated for the conditions in which the quantum dot labels were switched between the targeted and untargeted probes. Specifically, the staining cocktail was composed of QDAC-605-untargeted (conjugated to mouse IgG) and QDAC-655-targeted (conjugated to trastuzumab). All other conditions were maintained and the staining was performed at 37 °C. The resulting QDAC-DDSI images are provided in Fig 4(d) and ROC curve in Fig 5(d). The diagnostic performance of the DDSI parameter in this “reversed” pair condition was similar, though slightly higher than the other 37 °C staining conditions, with an AUC = 0.87. The DDSI images with corresponding H&E and HER2 IHC are provided in Fig 6(d), and show general qualitative correlation between DDSI and IHC of HER2.

## Discussion

To our knowledge, this study is the first to evaluate the use of a matched pair of receptor-targeted and untargeted quantum dot antibody conjugates to identify tumor and normal tissue. The results suggest that under certain conditions, the QDAC-DDSI technique can distinguish tumor and normal tissue with reasonably high diagnostic statistics (AUC > 0.81); however, the diagnostic performance was exceptionally sensitive to incubation temperature, with the AUC dropping to 0.61 for tissues stained at room temperature. Temperature is known to affect the rate of antibody-antigen binding [41], and this effect seems to be a primary driver of the QDAC probe kinetics in our protocol. Interestingly, this temperature sensitivity has not been observed in our extensive prior work which showed very high diagnostic performance using standard fluorophore labels [20, 37] for tissue staining at room temperature.

Temperature was the only condition which substantially impacted the diagnostic performance of the QDAC-DDSI technique, as all other conditions tested produced similar diagnostic performance. Specifically, the concentration of the staining solution did not significantly affect the diagnostic performance, as QDAC-DDSI using 100 nM and 10 nM staining solutions produced similar AUC metrics. Use of lower concentrations is important to minimize the amount of material needed and thus control procedure costs for QDAC-DDSI of larger specimens, such as encountered in BCS. Imaging QDAC’s with reversed labels showed significantly higher DDSI values compared to the other groups. This was not explicitly due to increased uptake of the QDAC’s on an absolute scale, but was largely a consequence of reduced uptake of the untargeted stain in both tumor and normal tissue and slightly elevated uptake targeted stain in tumor. This suggests that the label status had some effect on uptake, an issue the warrants further investigation. Nonetheless, the AUC performance remained relatively consistent, increasing by only 0.06 compared to the other 37° C staining conditions.

Consistent with our prior studies investigating tissue staining protocols for tumor diagnosis [20, 37], ROC analysis of the targeted QDAC probe alone showed no capacity to identify tumor and normal tissues. Thus, these results add to the expanding experimental evidence emphasizing the importance of incorporating the untargeted counterpart for fresh tissue staining. This dual-probe strategy effectively accommodates inhomogeneities in the imaging system and helps account for the confounding effects of non-specific uptake of the molecular probes. These corrected images are thus more representative of the biomarker-of-interest distribution.

Efforts to translate this strategy into clinical practice will require careful consideration of tissue processing time and workflow efficiency. In this initial feasibility study, the staining protocol used approximately 25 minutes to complete, which is too long for routine deployment in BCS. However, recent optimization efforts in our lab using our standard labels suggest that the time to complete the procedure can be reduce significantly without affecting the diagnostic performance [37]. In particular, recent data (not shown) suggest that a 1-minute blocking protocol provided similar diagnostic performance compared to longer blocking protocols (10 minutes) when using our standard organic-fluorophore-labeled antibody probes. Furthermore, staining times with the cyanine-labeled probes were reduced to 1 to 2 minutes for a total processing time of 6 minutes, which is far more reasonable for clinical translation. Future studies will examine these shortened protocols with QDACs.

Although our prior work with organic fluorophore labels resulted in higher AUC values compared to the QDAC-DDSI formulations used herein (in the same tumor line), there are several key advantages that would be enabled by the use of quantum dots and hyperspectral imaging for this application. The ability to excite several quantum dots with spectrally-distinct emission profiles using the same excitation source enables DDSI of multiple receptor targets simultaneously without increasing acquisition time. Additionally, this common excitation source ensures that the excited tissue volume is the same for all probes, eliminating the potentially confounding consequences that arise when different excitation sources excite different tissue volumes due to wavelength-dependent optical properties. These potential advantages compel further optimization and development of DDSI using QD-labeled antibodies.

This study adds to the growing body of literature examining the use of targeted and untargeted reference stains for rapid imaging of biomarkers in fresh specimens. These efforts have now shown this multi-agent approach effective in identifying tumor tissue with high accuracy using labeling strategies including SERS particles, conventional fluorophores and now quantum dots. Each of these strategies has associated advantages and drawbacks, and these trade-offs will require careful, application-specific consideration. Quantum dots and SERS particle labels are favorable for multiplexed imaging of multiple biomarkers with the same excitation source, which can be advantageous to minimize imaging time and to ensure consistent photon depth penetration between channels. Of the two, SERS provides the greatest capacity for multiplexing due to the sharp emission features compared with the other labels. The trade-off is imaging time, as current technology requires a raster-scanning acquisition implementation for SERS labels. Thus, imaging time and resolution become important trade-offs, especially for larger specimens, a challenge generally not associated with wide field imaging used with conventional and QD labels. Other parameters to consider include molecule size and charge, depth of penetration, cost, photostability, binding kinetics, etc., and the optimal strategy may be case specific. For example, diagnostic assessments requiring identification of tumor only in the most superficial layers would minimize requirements for stain and optical tissue penetration (in fact, these characteristics could be confounding). A full accounting of the performance trade-offs of these approaches will be an important consideration for each application.

## Conclusion

Rapid intra-operative assessment of margin status in surgical specimens to facilitate complete tumor removal remains an important but elusive capability. Emerging imaging strategies that use short-time-interval topical dual-probe correction techniques to emphasize the molecular differences between tumor and normal tissue have great promise for assessing margins of fresh specimens. In many ways, the unique properties of quantum dot labels, combined with hyperspectral imaging, make them ideal candidates for this modality, yet the diagnostic performance when using these quantum-dot complexes is particularly sensitive to staining conditions. The results herein are the first to demonstrate this approach using quantum dots and hyperspectral imaging, and establish a robust foundation for further development of this promising imaging strategy.

## Supporting information

S1 Appendix Flow cytometry cellular preparation. (DOCX)
