# Supplementary material for: Topical dual-probe staining using quantum dot-labeled antibodies for identifying tumor biomarkers in fresh specimens
Source: PLoS One. 2020 Mar 11;15(3):e0230267. doi: 10.1371/journal.pone.0230267 (PMC7065915; doi:10.1371/journal.pone.0230267)
Supplement: S1 Appendix — (DOCX) [file pone.0230267.s001.docx]

**Topical dual-probe staining using quantum dot-labeled antibodies for identifying tumor biomarkers in fresh specimens**

Boyu Meng^1^, Margaret R. Folaron^1^, Brook K. Byrd^1^, Kimberley S. Samkoe^2,3,4^, Rendall S. Strawbridge^1^, Connor Barth^5^, Summer L. Gibbs^5^ and Scott C. Davis^1,4,^ **^*^**

*^1^Thayer School of Engineering at Dartmouth College, 14 Engineering Dr. Hanover, NH, 03755*

*^2^Geisel School of Medicine at Dartmouth College, 1 Rope Ferry Rd, Hanover, NH 03755*

*^3^Department of Surgery, Dartmouth-Hitchcock Medical Center, 1 Medical Center Dr., Lebanon, NH 03756*

*^4^Norris Cotton Cancer Center, Dartmouth-Hitchcock Medical Center, 1 Medical Center Drive, Lebanon, NH 03756*

*^5^Biomedical Engineering Department, Oregon Health and Science University, Portland, OR, 972013*

**Short title: Tumor identification with quantum dot-based dual probe staining**

**Electronic Supplementary Information**

**†Corresponding author**

**Dr. Scott C. Davis**

**Thayer School of Engineering at Dartmouth College**

**14 Engineering Dr. Hanover, NH, 03755**

**Phone: (603) 646-9684**

**Email: Scott.C.Davis@dartmouth.edu**

**Flow cytometry cellular preparation**

Cells were trypsinized from T75 cell culture flasks, counted and placed in flow cytometry tubes at a seeding density of 1x10^6^, in triplicates per cell line. Next, the cells underwent a series of washes in phosphate-buffered saline (PBS) and blocking with 5% fetal bovine serum before incubation with Alexa Fluor 488-labeled trastuzumab for 1-2 hours. An additional PBS wash was performed, followed by fixation with 4% paraformaldehyde. Cells were washed and resuspended in PBS. The staining protocol was also performed on triplicates of both cell lines, excluding staining with Alexa Fluor 488-trastuzumab, in order to account for autofluorescence.
